# Transduction of Signals during Somatic Embryogenesis

**DOI:** 10.3390/plants11020178

**Published:** 2022-01-11

**Authors:** Mohamed Elhiti, Claudio Stasolla

**Affiliations:** 1 Department of Botany, Faculty of Science, Tanta University, Tanta 31527, Egypt; melhiti@farmersbusinessnetwork.com; 2 Department of Plant Science, University of Manitoba, Winnipeg, MB R3T2N2, Canada

**Keywords:** cell differentiation, epigenetic, growth regulators, phytoglobins (Pgbs), somatic embryogenesis, totipotency, transcription factors

## Abstract

Somatic embryogenesis (SE) is an in vitro biological process in which bipolar structures (somatic embryos) can be induced to form from somatic cells and regenerate into whole plants. Acquisition of the embryogenic potential in culture is initiated when some competent cells within the explants respond to inductive signals (mostly plant growth regulators, PRGs), and de-differentiate into embryogenic cells. Such cells, “canalized” into the embryogenic developmental pathway, are able to generate embryos comparable in structure and physiology to their in vivo counterparts. Genomic and transcriptomic studies have identified several pathways governing the initial stages of the embryogenic process. In this review, the authors emphasize the importance of the developmental signals required for the progression of embryo development, starting with the de-differentiation of somatic cells and culminating with tissue patterning during the formation of the embryo body. The action and interaction of PGRs are highlighted, along with the participation of master regulators, mostly transcription factors (TFs), and proteins involved in stress responses and the signal transduction required for the initiation of the embryogenic process.

## 1. Introduction

Plant embryogenesis starts with the fusion of the sperm cell with the egg, leading to the generation of the diploid zygote, which, through a coordinated cell division pattern, gives rise to a fully developed embryo [1]. This process can also be induced in culture where somatic cells (cells other than gametes) can be reprogrammed to embark into an embryonic developmental pathway leading to the formation of somatic embryos. Somatic embryogenic events are not uncommon in vivo under specific environmental circumstances, such as heat and drought. For example, Kalanchoë (*Kalanchoe delagoensis*) has been shown to spontaneously produce small bipolar structures its their leaves, and are then able to regenerate whole plants under suitable conditions [2]. The sporophytic apomictic developmental pathway is another example of embryogenesis (SE). This process occurs in the ovule and the embryos have the same genetic material as the mother plant (clones). Several reports also document the generation of embryos from microspores in culture, a process referred to as androgenesis [3]. Androgenesis requires a gametophytic–embryogenic transition with the subsequent formation of haploid embryos [4].

All the examples reported above highlight the fundamental concept of totipotency; that is, the inherent ability of plant cells to regenerate a whole plant through extensive reprogramming. Such reprogramming requires changes in gene expression, modifications of signaling networks, and the activation of specific regulatory pathways. As the initial step, competent cells of the cultured explant respond to inductive signals, inducing the de-differentiation step. Undifferentiated cells are subsequently “canalized” into embryogenic developmental pathways, culminating in the generation of embryos [5]. If the somatic embryos form directly on the cultured explant, the process is referred to as direct SE, while if the formation of the embryos is preceded by the proliferation of the explant cells and formation of an embryogenic tissue, it is referred to as indirect SE [6]. Many examples of direct or indirect SE are available in the literature [7].

Several inductive signals conducive to de-differentiation have been identified; they include plant growth regulators (PGRs) and heavy metals, as well as the imposition of stress conditions such as high temperature, osmotic shock, or water stress [8]. Understanding the cellular changes evoked by the inductive signals is very challenging, especially when two or multiple signals are required to initiate the process. An early attempt to document these changes using microarray studies revealed the involvement of genes encoding proteins related to hormone perception and response, as well as DNA methyltransferases and redox enzymes [3,5]. This study was followed by many others employing more novel techniques [9], which clearly highlighted the complexity of the de-differentiation step, and the difficulties in unequivocally identifying the key components participating in the response to the inductive signals.

A general consensus among tissue culturists is the requirement of auxins for the induction of the embryogenic process, as demonstrated by the use of this class of PGRs in many protocols [3]. The endogenous auxin level increases during the initial phases of embryogenesis [10], and this increase is linked to the activation of stress signals [11] and changes in chromatin status [12]. For example, profound changes in DNA methylation follow auxin application in culture medium, and some of these changes have been deemed as a requirement for the initiation of the embryogenic program [12]. The requirement for auxins is transient and specific to the initial stages of embryogenesis, which are often characterized by the formation of the embryogenic tissue; the subsequent phases can occur in an auxin-free environment. This general notion is applicable to many species [9,13], including *Arabidopsis*, which is the model system in plant biology.

## 2. Somatic Embryogenic Systems: *Arabidopsis* as a Model

Somatic embryogenesis (SE) represents a valuable tool to study the developmental aspects of in vivo plant embryogenesis for a number of reasons. First, in vitro and in vivo embryogenesis share many structural, physiological, biochemical, and molecular similarities [14]. This is an important characteristic given the difficulties in studying early embryogeny; in vivo embryos reside inside the maternal tissue and are difficult to dissect [1,5]. Secondly, many of the SE systems currently available form embryos in a synchronous fashion, thus facilitating stage-specific analyses. This concept is best exemplified in carrot, spruce, alfalfa, and cotton systems, some of which have provided a wealth of information related to plant embryogenesis [15]. In the past twenty years, the optimization of in vitro embryogenic protocols in *Arabidopsis* [16] has allowed the integration of genetic studies [17] that would have been impossible to conduct in other species [5,18,19,20,21]. For example, the use of mutants has highlighted the existence of master regulators modulating auxin responses [18] and of signal molecules [5] required for the execution of SE.

A wide range of explants can be used to initiate the embryogenic path in *Arabidopsis*, including immature zygotic embryos [5,22], mature zygotic embryos (dry seeds) [23], leaf protoplasts [24], as well as shoot apices and flower buds [25]. In all protocols, the embryogenic process is initiated by the synthetic auxin, 2,4-dichlorophenoxyacetic acid (2,4-D), although heavy metal, salt, or osmotic stress treatments, alone or in combination with 2,4-D, can also be used [25]. Moreover, the primary somatic embryos induced by 2,4-D treatments can further produce secondary embryos via embryogenic callus [22,26], a system that allows the production of more embryos.

Despite some variations depending on the source of the original explant, somatic embryogenesis encompasses two steps: an induction phase followed by a development phase. The induction phase, lasting about 14 days and requiring 2,4-D, is needed for the generation of the embryogenic tissue, composed of immature somatic embryos. The continuation of embryo growth is encouraged in the hormone-free development medium where developed embryos become visible after 9 days. This system can be direct or indirect, i.e., requiring the formation of an intervening callus phase, and can occur on solid or liquid media [16]. Caution must be used when using the *Arabidopsis* somatic embryogenic system as both somatic embryos (bipolar in their structure) and adventitious shoots often form from the same explant [27]. In the same study it was demonstrated that somatic embryos and adventitious shoots do not share a similar ontology. While shoots are attached to the explant though a wide tissue base, somatic embryos develop from isolated cell clusters and are connected by a narrower connection. Independent studies suggest that adventitious shoot and somatic embryo formation represents a developmental continuum, and their respective formation is due to differing levels of auxin [28].

### 2.1. Roles of Plant Growth Regulators (PGRs) during SE

Plant growth regulators (PGRs), key chemical substances governing plant growth and development, are also required during embryogenesis. Among the different PGRs, auxin is perhaps the most well characterized in relation to embryo development in vivo owing to its requirement in the establishment of an apical–basal axis and radial symmetry [29]. During early in vivo embryogenesis, the expression of PIN1 and PIN7 is coincidental to the induction of auxin response occurring in the two-celled embryo [30]. Here, the flow of auxin moves acropetally to the apical cell through PIN7. This pattern, continuing until the 32-cell stage of embryogenesis, is needed for the specification of the shoot apical meristem. During the following stages, PIN1 regulates the basipetal movement of auxin needed for the establishment of the root apical meristem, and mediates the translocation of auxin towards the incipient cotyledon primordia. Low auxin environments at the shoot tip define the stem cells, the regulation of which relies on the well characterized WUSCHEL-CLAVATA signaling [31]. This unique and conserved localization pattern ensures fluxes and maxima of auxin that specify cell fate in the developing embryos. Perturbations in the auxin movement compromise embryogenesis, as observed in *pin7* and *pin1* mutants [30].

Auxin is also critical during in vitro embryogenesis, where it acts as the signal required for the induction and proliferation of embryogenic tissue [5,26,32,33,34,35]. While in some systems, including *Arabidopsis*, the application of auxins is only needed for the formation of the embryos, but not their subsequent development [30]; in others, the transition from immature to mature embryos also necessitates auxins [36]. It must be noted that the inhibition of embryogenesis by auxins has sometimes been reported, such in the case of *Abies nordmanniana* [37], an observation consistent with the specific requirements for different species. Combinations of different auxins, such as 2,4-D and NAA, or auxins with cytokinins have also been used to stimulate the embryogenic process [38]. Exogenous applications of auxins induce a rise in its endogenous content, which translates to cell reprogramming [5,39].

Besides auxins, cytokinins (CKs) are also key regulators in a variety of embryogenic systems. In vivo, cytokinin promotes the formation of buds and, with auxins, stimulates cell division [37]. It is well recognized that a high CK:auxin ratio induces the production of shoots, while a low ratio generates roots; this notion is applied in many propagation protocols [40]. By using the DR5 reporter for auxin and a two-component system (TCSv2) for CKs, [39] demonstrated the interactive role of these two PGRs. It was also demonstrated that CKs regulate the synthesis of auxin during the formation of shoots and roots [40]. It is plausible that a similar mechanism occurs in those embryogenic systems where CKs are required in conjunction with auxins to stimulate embryo development.

While CKs and auxins are mainly needed at the onset of the SE process, ABA is often required to sustain embryo growth [41,42]. This is best exemplified in spruce, where the removal of auxins and the inclusion of ABA in the culture medium are required steps for the continuation of embryo development [43]. The role of ABA during somatic embryogenesis is similar to that described in vivo, where this PGR is implicated in the accumulation of storage products and confers tolerance to desiccation during the late stages of maturation [44]. In recent studies, the function of ABA synthesis and signaling during the in vitro embryogenic process has been analyzed, with *ABSCISIC ACID INSENSITIVE 3* (*ABI3*) and *ABI4* transcription factors shown to play a relevant role in embryo formation [42].

Besides ABA, another stress hormone, ethylene, is also involved in SE, as demonstrated by the requirement for 1-aminocyclopropane-1-carboxylic acid (ACC), the ethylene precursor, during the early phases of embryogenesis [45]. However, the overaccumulation of this PGR can be deleterious for the development of the embryos, as reported by Kong and Yeung (1995), who documented the deterioration of the shoot apical meristem due to ethylene [46]. In the same study, it was reported that pharmacological treatments suppressing ethylene synthesis restored the integrity of the embryos.

Besides the classical PGRs, recent studies suggest that jasmonic acid also contributes to the formation of somatic embryos, further linking the embryogenic process to stress signaling. Network enrichment analyses during *Pseudotsuga menziesii* SE highlighted the relevance of proteins participating in the metabolism of jasmonic acid [47]. Produced in vivo in response to conditions of biotic and abiotic stress, jasmonic acid has been shown to promote microspores embryogenesis and enhances the quality of microspore-derived embryos in *Brassica* [48]. This effect was also reported in other species such as *Medicago* [49] and *Nicotiana* [50]. Jasmonic acid also prevents precocious germination in *Brassica*, and these effects are comparable to those observed for ABA [51]. A more recent study integrated jasmonic acid to auxin and nitric oxide (NO) in *Arabidopsis* SE [52]. The authors observed that conditions elevating the levels of NO through suppression of the NO scavenging protein phytoglobin 2 (*Pgb2*) and the levels of jasmonic acid, which stimulates auxin synthesis and the formation of the somatic embryos. An intermediate linking jasmonic acid and auxin was identified as MYC2, a key component in the regulation of stress responses in vivo.

It is apparent from the studies reported above that auxin has received most of the attention given its role in cellular de-differentiation, which is an obligatory step in any in vitro embryogenic processes. As such, the following section will examine in depth the function of this PGR during embryogenesis.

### 2.2. Auxin Responses during SE

The auxin-mediated transition of somatic cells into embryonic cells is accompanied by profound transcription changes [53]. Genes most affected by auxin fall into four major categories: transcription factors (TFs), cell cycle regulators, enzymes participating in the biosynthesis of other PGRs, and components of diverse cellular signal transduction pathways [5]. Of note, the exogenous application of auxin has been linked to its endogenous increase, as revealed by the upregulation of several biosynthetic enzymes, such as *TRYPTOPHAN AMINOTRANSFERASE OF ARABIDOPSIS* 1, YUCCA1, and YUCCA 3 [54]. This increment is accompanied by a specific auxin distribution pattern within the explant, which reflects the PIN-mediated auxin dynamics observed during in vivo embryogenesis [26] as described in the previous section.

The establishment of defined auxin gradients also acts as developmental signals during SE [26]. The PIN1-mediated movement of auxin in *Arabidopsis* explants observed after only 16 h in induction medium contributes to the delineation of the WUSCHEL-expressing cells that define the future sites of embryo formation. Pharmacological treatments perturbing auxin movement preclude these events and lower the number of somatic embryos [24]. A similar requirement for auxin gradients during SE was also reported by Elhiti et al. (2013) who observed the specific localization patterns of both PIN1 and PIN2 during the early phases of *Arabidopsis* embryogenesis [55]. These patterns established auxin maxima in the cotyledons of the zygotic embryos used as explants and contributed to the generation of the embryogenic tissue and ultimately increased the number of somatic embryos [55].

Auxin activates signal transduction pathways with *AUXIN RESPONSE FACTORs* (*ARFs*) controlling many responses [56]. In total, 23 *ARFs* have been identified in *Arabidopsis* [57], some of which participate in embryogenic processes [58]. A comprehensive analysis revealed differential expression patterns of *ARFs* during the initial stages of Arabidopsis SE: six *ARFs* (*ARF5*, *ARF6*, *ARF8*, *ARF10*, *ARF16*, and *ARF17*) were significantly upregulated during the induction phase, whereas five (*ARF1*, *ARF2*, *ARF3*, *ARF11*, and *ARF18*) were substantially downregulated [59]. The mechanisms by which auxin regulates gene expression have been partially characterized. Without auxin, the Aux/IAA protein interacts with ARF, inhibiting its activity and depressing the auxin response. When auxin, is present the Aux/IAA protein is targeted for degradation by the SKP-Cullin-F-boxTIR1/AFB (SCFTIR1/AFBs) E3 ubiquitin ligase complex. It has been shown that several AUX/IAA mutants, including IAA16, 29, 30, and 31, have negative effects on SE [60].

### 2.3. Stress Signaling during SE

#### 2.3.1. Wounding Stress

The SE process is initiated with the dissection and culturing of explants, and during this process, cellular damage and wounding are inevitable. The contribution of stress responses to SE has been suggested, with reactive oxygen species (ROS) playing a fundamental role [25,61]. It has been reported that ROS are produced a few hours after wounding [62]. Moreover, treatments with ROS also promote the formation of somatic embryos. For example, the inclusion of hydrogen peroxide augments the number of somatic embryos in *Lycium barbarum* [63]. In agreement with the requirement of ROS, the depletion of ROS inhibits the ability to form embryos. The ROS inhibitor, diphenyleneiodonium (DPI), suppresses embryogenesis in many species, including alfalfa, *Arabidopsis*, and tobacco [64]. Collectively, these results support the requirement for ROS during SE.

Besides ROS, other stress-related factors induced by wounding might also contribute to the initiation of the embryogenic process. For examples, several genes encoding cell wall re-modelling factors, such as expansin, extensin, pectinesterase, and glucanase, are upregulated at the onset of wounding [65]. The modification of cell wall components has been linked to cell fate acquisition [66]. Genes participating in wounding responses might play a relevant role during SE. For example, *WOUND INDUCED DEDIFFERENTIATION 1* (*WIND1*) is involved in the acquisition and retention of the de-differentiation status of somatic cells following wounding [67]. Its overexpression generates callus-like clusters around the shoot meristem, and SE [68].

#### 2.3.2. Osmotic Stress

Osmotic stress, created by a variety of osmotic agents or as a result of a saline environment, can often promote embryogenesis. In the *Triticum aestivum* SE system, the application of 40 mM NaCl in culture medium increases the number of somatic embryos [69], while elevated sucrose levels are sufficient to generate somatic embryos from cultured shoot segments of carrot [70]. These treatments are also known to increase the production of ROS, which, as explained above, can be conducive to the development of somatic embryos [71]. Commonly used osmotic agents are polyethylene glycol and betaine. Polyethylene glycol improves date palm SE [72] and is routinely employed to enhance embryo number and desiccation tolerance in coniferous species [43]. Betaine enhances SE in tea [73]. Water stress, which has beneficial effects on the production of somatic embryos, can also be achieved by choosing the right concentration of gelling agent [74]. While the effects of low osmoticum are not fully understood, it is apparent that water limitation can induce cellular changes favoring the embryogenic and conversion processes. Recently, Valencia-Lozano et al. (2021) showed that 9.0 g/L gelrite (−1.47 MPa) enhanced the conversion of *Coffea arabica* somatic embryos from 39% to 95% [75]. A manipulation of sucrose levels can also influence SE [25].

#### 2.3.3. Temperature Stress

Temperature stress is also known to regulate in vitro embryogenesis, as it is apparent in *Brassica* where microspore donor plants are subjected to 12–15 °C Day/7–10 °C night prior to the collection of the spores, which are subsequently incubated at 32 °C for 8–72 h [76]. The latter heat shock is not only required to trigger the gametophytic–embryogenic transition of the microspores [5], but also to regulate tissue pattering. Joosen et al. (2007) and Dubas et al. (2014) demonstrated that the heat shock treatment for 24 h was linked to the production of suspensors, which was not observed for longer treatments [77,78]. These divergent responses were attributed to the effect of the length of the temperature regime to the endogenous levels of auxin, which is known to control the apical–basal polarity of the embryos [78]. *Brassica* is not the only system responsive to temperature stress. In the *Cichorium intybus* × *Cichorium endivia* system, direct SE was induced when the explants were incubated at 35 °C, while shoot regeneration was stimulated at 25 °C [79]. Similarly, Touraev et al. (1996) observed an increased number of tobacco somatic embryos from shoot tips following the imposition of high temperatures, ranging from 33 °C to 37 °C [80]. Recently, Castander-Olarieta et al. (2021) reported that the exposure of *Pinus radiata* explants to 60 °C for 5 min significantly promotes embryogenic competence [81]. These effects were associated with an elevated abundance of proteins involved in the post-transcriptional regulation (ARGONAUTE 1D), as well as biosynthesis of fatty acids, sugars, and cell wall carbohydrates.

## 3. Programmed Cell Death (PCD) during SE

Programmed cell death (PCD) is a conserved process occurring during plant embryogenesis, which is responsible for the dismantling of the suspensor and the removal of subordinate embryos in gymnosperm seeds [81,82]. During in vitro embryogenesis PCD eliminates specific embryogenic cells [83], and it is required for shaping the embryo body, as unequivocally demonstrated in spruce [84]. In this system, pro-embryogenic masses (PEMs), originating from zygotic embryos, are maintained in a medium containing auxin and cytokinin [85]. Pro-embryogenic masses I (PEM I) are composed of a cluster of cytoplasmic cells attached to a single suspensor cell. The addition of another suspensor cell characterizes the PEMII, and as more suspensors are added, PEMII transition into PEMIII.

With the inclusion of plant growth regulators (PGRs), the three PEMs retain their structure. The formation of immature somatic embryos from PEMIII is stimulated by removing PGRs, and this process requires PCD [84]. Pharmacological treatments abolishing PCD preclude the differentiation of PEMIII into somatic embryos [86], thus demonstrating the obligatory requirement of the death program for SE. Huang et al. (2014) demonstrated that the accumulation of nitric oxide (NO) in cells destined to die precedes the execution of PCD and this effect is mediated by the accumulation of reactive oxygen species (ROS) [86]. Factors suppressing NO or ROS reduce PCD and suppress embryo formation [82,86]. A second wave of PCD involves the degradation of the suspensor cells in the somatic embryos during the later stages of development [84]. The induction of programmed cell death in spruce is regulated by metacaspases and is characterized by cellular features related to apoptotic events observed in animal systems [87].

The execution of PCD is also a critical component of microspore cultures, especially during the early stages of embryogenesis [80]. In *Brassica*, two PCD events were observed. The first wave contributes to the degeneration of the tapetum cells within the anthers during pre-meiosis [88]. This is needed to redirect cell development toward the embryogenic state [89]. An additional PCD event accompanies the differentiation of multicellular aggregates (formed by regions originating from the generative cell and the vegetative cell) into embryo-like structures. The elimination of the generative domain promotes the development of the vegetative domain into embryo-like structures [82,86]. Based on the above research, it is obvious that PCD is needed for the formation of embryos, and understanding the factors underlying PCD is critical to manipulate the embryogenic process.

## 4. Translation of Stress Signals during SE

Many stress-related genes are differentially expressed at the onset of embryogenesis in a cell-specific fashion [90]. Among these genes are heat shock components and several hydrolytic enzymes, including nucleases, proteases, and glucosidases, which are induced during different stages of embryo development [91]. In potato, for example, an increase in the expression of many stress-related genes coincided with the induction of SE, and this was followed by an elevation in oxidative stress [91]. Thibaud-Nissen et al. (2003) observed that genes involved in the oxidative burst were upregulated during the first 14 days of *Glycine max* SE in the presence of 2,4-D, including many *GLUTATHIONE-S-TRANSFERASE* genes (*GST7*, *GST8*, *GST11*, *GST16*, and *GST19*) [65]. These results were confirmed by Elhiti et al. (2012), who also observed the activation of antioxidant responses, including ROS-detoxifying enzymes such as catalases, superoxide dismutases, and components of the Halliwell-Asada cycle [18]. The role of antioxidants as potential regulators of SE was demonstrated by an early study documenting the beneficial effect of ascorbic acid and glutathione during spruce and *Brassica* in vitro embryogenesis [18]. Other plant defense and stress-related genes highly induced within the first 24 h of SE are *WOUND INDUCED PROTEIN 1* (*WIP1*) and *CHITINASE A1* [92]. It has also been reported that SE-related genes, such as *AGAMOUS-15* (*AGL15*) [93] and *SOMATIC EMBRYOGENESIS RECEPTOR KINASE1* (*SERK1*) [4], are induced in *Arabidopsis* by stress. ABA signaling is an important transducer of stress responses and *ABA2* (short-chain alcohol dehydrogenase) was highly upregulated during cotton SE alongside with jasmonic acid-related genes [90]. The participation of PGRs in wounding responses was also confirmed in a time-course experiment conducted during maize SE [94].

## 5. Transcription Factors and Signal Transduction

Independent studies suggest that components involved in transcription and signal transduction play a fundamental role during in vitro embryogenesis. The following section analyses the effects of some of these components on SE.

### 5.1. SHOOT MERISTEMLESS (STM)

*SHOOT MERISTEMLESS* (*STM*) is a homeobox gene, the product of which is a member of the class-1 KNOX homeodomain-containing proteins [95]. These proteins are present in the shoot apical pole where they regulate the behavior of the meristematic cells [96]. *STM* is initially detected in a few cells of immature embryos in *Arabidopsis*, and then extends to larger apical domains [97]. Genetic and molecular analyses revealed that *STM* suppresses MYB-related genes, such as *ASYMETRIC LEAVES 1* (*AS1*), which are required to initiate organogenesis [98]. It has been reported that ectopic expression of *STM* modulates the sensitivity of the tissue to exogenous auxin [5]. This might be due to either an increased sensitivity to 2,4-D and/or a higher endogenous auxin level. Moreover, proper levels and distributions of auxin would increase *WUS* expression, which is known to specify embryogenic cell fate [26]. The overexpression of *STM* during SE regulates the expression of many genes participating in hormone synthesis and signaling, as well as genes encoding DNA methyltransferases and components of glutathione metabolism [5]. The effect of STM overexpression on chromatin modification was documented, with pharmacological treatments demonstrating that global hypomethylation of DNA during the induction phase encourages the embryogenic process in *Brassica* [5].

### 5.2. WUSCHEL (WUS)

*WUSCHEL* (*WUS*) is a homeobox gene required for the formation and maintenance of the “organizing center” of the shoot apical meristem [99]. By acting as a repressor of other factors [100], WUS is needed for the retention of pluripotency and “stemness” in the shoot apical meristem [101]. An important characteristic of WUS is its ability to cross the cell layers of the apical pole [101] where it promotes the transcription of CLAVATA3 (CVL3) [102]. The WUS-CLV feedback loop is required for the maintenance of the apical meristem [103].

When ectopically expressed, *WUS* induces the de-differentiation of somatic cells and the subsequent generation of adventitious shoots and somatic embryos in several species, including *Arabidopsis* [104] *Nicotiana tabacum* [105], *Coffea canephora* [106], *Gossypium hirsutum* [107], *Sorghum bicolor*, and *Zea mays* [108]. These studies suggest a conserved function of WUS across species. It has been reported that *WUS* responds to endogenous auxins in explant cells [26]. The auxin vegetative-to-embryogenic transition is in fact mediated by the expression of *WUS* [104]. Specifically, the establishment of auxin maxima correlates with the induction of *WUS* expression during the initial phases of SE [26]. In *Coffea canephora*, the overexpression of *WUS* promotes SE in heterologous systems [106] and consequently increases the number of somatic embryos by 400%. The promotive effect of the ectopic expression of *AtWUS* on SE coincides with the upregulation of other transcription factors, i.e., *LEC1*, *LEC2*, and *FUS3*, known to regulate key developmental aspects of plant development [104,107].

### 5.3. LEAFY COTYLEDON (LEC)

*LEAFY COTYLEDON* (*LEC*) genes are crucial in the regulation of plant embryogenesis [109]. While *LEC1* encodes a protein similar to the HAP3 subunit of the CCAAT binding factor [110], *LEC2* is similar to the B3 domain, which is a DNA-binding motif typical of transcription factors, participating in the development of seeds [111]. The ectopic expression of *Arabidopsis* *LEC1* and *LEC2* is sufficient to promote the vegetative embryogenic transition and induce the formation of somatic embryos [19,110]. In conifers, *LEC1* transcripts are present only in pro-embryonic masses (PEMs) and not in non-embryogenic tissue [112]. In pine, the overexpression of the *LEC1*-type gene (*PaHAP3A*) stimulates the formation of secondary somatic embryos [112]. The overexpression of *LEC2* has also been exploited to enhance SE, as demonstrated in *Theobroma cacao* [113], and these effects were linked to the induction of FUS3, ABI3, and WRI1 [114], as well as an increase in auxin level [115]. A study conducted by Brand et al. (2019) suggests that *LEC1* and 2 might activate slightly different responses, with *LEC1* encouraging the formation embryogenic tissue, and L*EC2* the direct formation of somatic embryos [116].

### 5.4. BABY BOOM (BBM)

*BABY BOOM* (*BBM*) encodes an AINTEGUMENTA-LIKE (AIL) APETALA2/ethylene-responsive element-binding factor (AP2/ERF). It is involved in several processes ranging from cell division to general aspects of plant development [117]. In *Arabidopsis*, *AIL* genes are a small cluster within the AP2/ERF transcription factor family. This family also includes *AINTEGUMENTA* (*ANT*), *AIL1*, *PLETHORA1* (*PLT1*), *PLT2*, *AIL6/PLT3*, *CHOTTO1* (*CHO1*)/*EMBRYOMAKER* (*EMK*)/*AIL5*/*PLT5*, *PLT7*, and *BBM* [13]. *AIL* genes are present in rapidly dividing tissues and are also involved in the maintenance of meristematic identity [13]. This is best exemplified by multiple *AIL* knockout mutants that exhibit reduced cell proliferation, altered cell identity, aberrations in floral development [118], and embryo defects [119].

The induction of SE by *BBM* occurs in a dose-dependent fashion and through the regulation of several transcription factors [13]. In *Arabidopsis thaliana* and *Brassica napus*, *BBM* changes in spatial/temporal expression during embryogenesis [120,121]. It is expressed in the pro-vascular tissue of heart-staged embryos and in the stem cells of the root apical meristem [119]. Considered as a “biomarker” of embryogenesis [122], BBM facilitates the acquisition of embryogenic fate and induces spontaneous somatic embryos in *Arabidopsis thaliana* and *Brassica napus* [117,121], and recalcitrant species [13]. The heterologous expression of *BBM* from *Arabidopsis thaliana* and *Brassica napus* in *Nicotiana tabacum* also results in an increase in the regeneration potential [123]. Relevant effects of this gene on the in vitro embryogenic process have been described in many studies [122,124,125,126,127].

### 5.5. SOMATIC EMBRYOGENESIS RECEPTOR KINASES (SERK)

Somatic embryogenesis receptor-like kinase (SERK) belongs to a cluster of proteins of the subgroup II of receptor-like kinases (RLK). The first SERK gene, *DcSERK*, was isolated from a cDNA library from carrot embryogenic cell cultures [128]. In *Arabidopsis thaliana*, five SERK homologs have been identified, *AtSERK1*–*AtSERK5* [129]. Among the different SERKs, SERK1 is highly expressed in embryogenic cultures and can be used as a reliable marker for competence to SE. During *Arabidopsis* SE, *SERK1* is highly present in all the embryogenic cells and developing embryos up to the heart stage of development, and its overexpression promotes the formation of somatic embryos [129]. These effects of SERKs on SE have been reported in more than one species [130] and have been linked to auxin signaling and the ability to confer pluripotency [130]. The ectopic expression of SERK1 favors the induction of somatic embryos by conferring embryogenic competence [129], and its expression level can be used to differentiate embryogenic vs. non embryogenic cells [131,132]. Plant hormones play an important role in SERK expression and responses. In *Medicago truncatula*, the expression of SERK1 is stimulated by auxins and CKs in a synergistic fashion [130]. While SERK2 and SERK3 elicit auxin-specific responses, SERK1 and SERK5 are interconnected with brassinosteroid signaling [132]. Overall, it is well recognized that SERK genes are involved in the regulation of plant totipotency and pluripotency.

### 5.6. Phytoglobins (Pgbs)

Phytoglobins (Pgbs) are heme-containing proteins known for their participation in stress-related responses through their ability to scavenge NO [133,134]. Phytoglobins have also been reported to influence in vitro embryogenesis in both dicots and monocots. In *Arabidopsis*, the repression of *Pgb2* promotes the generation of embryogenic cells from the cotyledons of the zygotic embryos used as explants [61]. The authors proposed that a reduction in *Pgb2* level increases NO, which is a repressor of *MYC2*. MYC2 is a stress-induced transcription factor suppressing the synthesis of auxin [61]. The elevation of auxin promotes the formation of the embryogenic tissue and the expression of WUS [26]. This model was further elaborated by the work of Mira et al. (2017) on integrating jasmonic acid signaling in the elevation of auxin level by NO [135].

In maize, the effects of Pgbs on SE are linked to their ability to regulate PCD. The suppression of *ZmPgb1.1* or *ZmPgb1.2* promotes PCD through the activation of cellular NO, which favors the release of Zn^2+^ from metallothioneins. Increasing levels of Zn^2+^ induce the mitogen-activated protein kinase (MAPK) cascade, with a subsequent increment in cellular ROS and activation of PCD [86]. Although both *ZmPgb1.1* and *ZmPgb1.2* operate through identical mechanisms, they have opposite effects on SE: the suppression of *ZmPgb1.1* reduces the number of somatic embryos, while the suppression of *ZmPgb1.2* enhances the embryogenic process [86]. These contrasting effects are due to the expression patters of the two genes. *ZmPgb1.2* is present in a few cells attaching the immature somatic embryos to the embryogenic tissue. Therefore, the dismantling of these by PCD, when *ZmPgb1.2* is downregulated, frees the embryos, which can proceed through their development. In contrast, *ZmPgb1.1* is expressed in many embryonic cells and the suppression of this gene causes extensive PCD and embryo abortion [86]. The ability to target Pgbs in specific cells can thus contribute to changes in cell behavior and ameliorate SE.

## 6. Adaptor Proteins

Recently, adaptor proteins have been shown to be important during the induction of somatic embryos [17,61]. 14-3-3 adaptor proteins participate in the signal transduction pathway shared by several PGRs. In *planta*, the number of adaptor proteins is species dependent: 13 in *Arabidopsis* [136], 6 in cotton [137], 17 in tobacco [138], 10 in tomato [139], 5 in barley [140], and 8 in rice [141]. The functions of 14-3-3 adaptor proteins have been associated with auxin transport [142] and SE induction [143]. Embryogenic cultures of *Cyclamen persicum* have higher levels of 14-3-3-like protein relative to their non-embryogenic counterparts. The adaptor protein phosphatase 2A (PP2A) is present in embryogenic tissue [144]. This protein has three components: a catalytic subunit, a regulatory A subunit, and a variable B subunit [145]. The A subunit, essential for auxin transport, has been associated with the SE process [146]. The differences in phosphorylation levels differentiating embryogenic tissue from non-embryogenic tissue has also been linked to the levels of PP2A [142]. Based on this very preliminary evidence, it is suggested that adaptor proteins might regulate SE through the mediation of auxin.

## 7. Concluding Remarks

**Figure 1:**
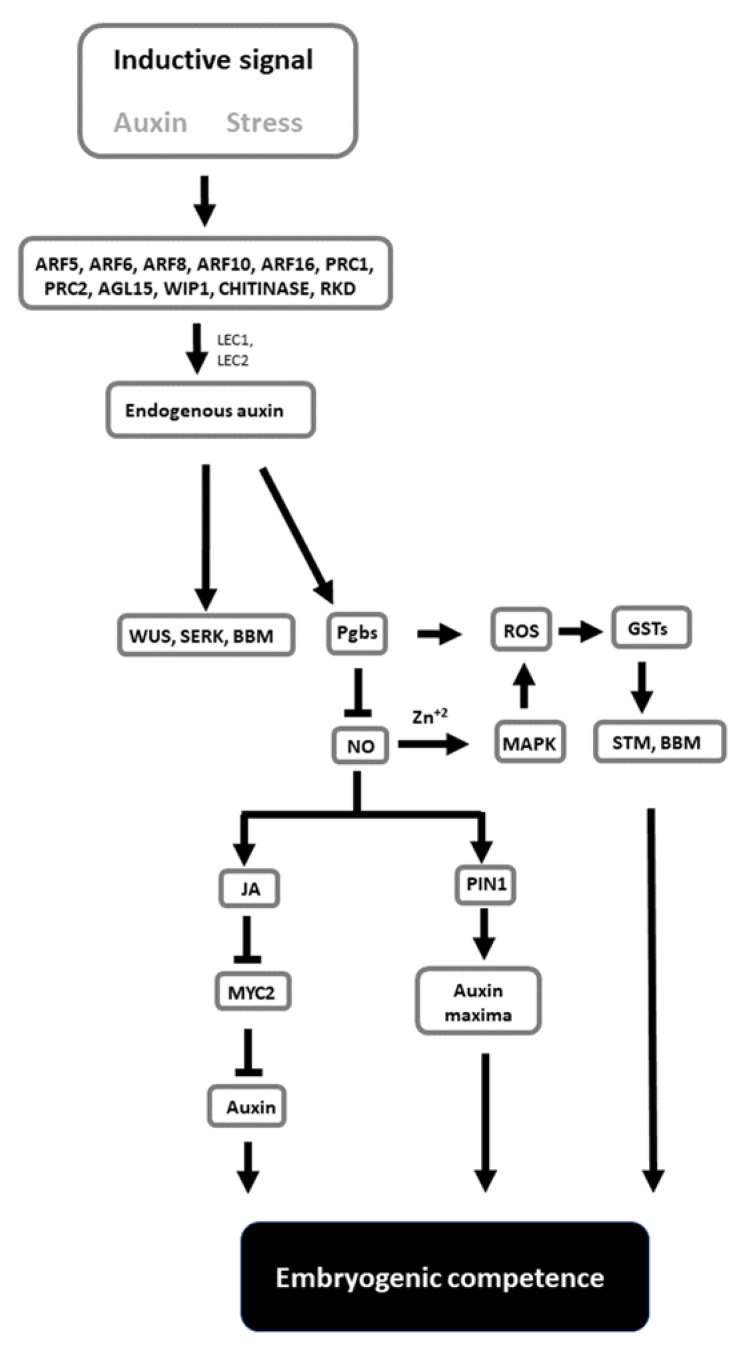
Tentative model highlighting the relevant components contributing to the acquisition of embryogenic competence. Inductive signals trigger responses increasing the level of endogenous auxin, which promotes the expression of SE marker genes such as WUS, SERK and BBM. Auxin also induces Pgbs, which, through suppression of NO, initiate a cascade of events mediated by JA and MYC2 and culminating with changes in auxin level and localization. The process also requires oxidative responses. Genes indicated in the figure are described in more detail in the text.

Despite the complexity of events associated with the formation of embryos in culture, SE has the potential to be used as a model to unravel the mechanisms governing totipotency and pluripotency. The combined applications of genetic knowledge derived from *Arabidopsis*, as well as the ability to alter the composition of the culture medium and growth environment, represent versatile and viable options to study cell behavior. The information provided in this review is conceptualized in a model (Figure 1). In this model, the inductive signals linked to stress and mediated by auxin activate a cascade of genes, resulting in diverse responses. Some of these responses cause changes in the endogenous auxin levels conducive to the induction of SE marker genes (WUS, SERK, and BBM) and cell totipotency. Auxin also modulates Pgbs, which, through the suppression of NO and the mediation of JA and auxin transport, regulates embryogenic competence. This process integrates ROS and oxidative responses. While extremely speculative, this model can be used as a framework that should encourage further studies examining how the proposed signals needed to execute SE can be integrated in a broader model linking molecular events to changes in cell fate.

## Data Availability

Data is contained within the article.

## References

[1] Willemsen V., Scheres B. (2004). Mechanisms of pattern formation in plant embryogenesis. Annu. Rev. Genet..

[2] Garcês H.M.P., Sinha N. (2009). The ‘mother of thousands’ (*Kalanchoë daigremontiana*): A plant model for asexual reproduction and CAM studies. Cold Spring Harb. Protoc..

[3] Karami O., Philipsen C., Rahimi A., Nurillah A.R., Boutilier K., Offringa R. (2021). Endogenous auxin directs development of embryonic stem cells into somatic proembryos in *Arabidopsis*. bioRxiv.

[4] Radoeva T., Weijers D. (2014). A roadmap to embryo identity in plants. Trends Plant Sci..

[5] Elhiti M., Tahir M., Gulden R.H., Khamiss K., Stasolla C. (2010). Modulation of embryo-forming capacity in culture through the expression of *Brassica* genes involved in the regulation of the shoot apical meristem. J Exp. Bot..

[6] Quiroz-Figueroa F.R., Monforte-González M., Galaz-Ávalos R.M., Loyola-Vargas V.M., Loyola-Vargas V.M., Vázquez-Flota F.A. (2006). Direct somatic embryogenesis in *Coffea canephora*. Plant Cell Culture Protocols.

[7] Hazubska-Przybył T., Ratajczak E., Obarska A., Pers-Kamczyc E. (2020). Different roles of auxins in somatic embryogenesis efficiency in two *Picea* species. Int. J. Mol. Sci..

[8] Nic-Can G.I., Loyola-Vargas V.M., Loyola-Vargas V.M., Ochoa-Alejo N. (2016). The role of the auxins during somatic embryogenesis. Somatic Embryogenesis: Fundamental Aspects and Applications.

[9] Ji L., Mathioni S.M., Johnson S., Tucker D., Bewick A.J., Do Kim K., Daron J., Slotkin R.K., Jackson S.A., Parrott W.A. (2019). Genome-wide reinforcement of DNA methylation occurs during somatic embryogenesis in soybean. Plant Cell.

[10] Ribnicky D.M., Cohen J.D., Hu W.S., Cooke T.J. (2002). An auxin surge following fertilization in carrots: A mechanism for regulating plant totipotency. Planta.

[11] Feher A., Pasternak T.P., Dudits D. (2003). Transition of somatic plant cells to an embryogenic state. Plant Cell Tissue Organ Cult..

[12] Phillips R.L., Kaeppler S.M., Olhoft P. (1994). Genetic instability of plant tissue cultures: Breakdown of normal controls. Proc. Natl. Acad. Sci. USA.

[13] Horstman A., Li M., Heidmann I., Weemen M., Chen B., Muiño J.M., Angenent G.C., Boutilier K. (2017). The *BABY BOOM* transcription factor activates the LEC1-ABI3-FUS3-LEC2 network to induce somatic embryogenesis. Plant Physiol..

[14] Zimmerman J.L. (1993). Somatic Embryogenesis: A Model for early development in higher plants. Plant Cell.

[15] Zavattieri M.A., Frederico A.M., Lima M., Sabino R., Arnholdt-Schmitt B. (2010). Induction of somatic embryogenesis as an example of stress-related plant reactions. Electron. J. Biotechnol..

[16] Mordhorst A.P., Voerman K.J., Hartog M.V., Meijer E.A., van Went J., Koornneef M., de Vries S.C. (1998). Somatic embryogenesis in *Arabidopsis thaliana* is facilitated by mutations in genes repressing meristematic cell divisions. Genetics.

[17] Horstman A., Bemer M., Boutilier K. (2017). A transcriptional view on somatic embryogenesis. Regeneration..

[18] Elhiti M., Yang C., Belmonte M.F., Gulden R.H., Stasolla C. (2012). Transcriptional changes of antioxidant responses, hormone signalling and developmental processes evoked by the *Brassica napus* SHOOTMERISTEMLESS during in vitro embryogenesis. Plant Physiol. Biochem..

[19] Stone S.L., Braybrook S.A., Paula S.L., Kwong L.W., Meuser J., Pelletier J., Hsieh T.F., Fischer R.L., Goldberg R.B., Harada J.H. (2008). *Arabidopsis LEAFY COTYLEDON2* induces maturation traits and auxin activity: Implications for somatic embryogenesis. Proc. Natl. Acad. Sci. USA.

[20] Tanaka M., Kikuchi A., Kamada H. (2008). The *Arabidopsis* histone deacetylases HDA6 and HDA19 contribute to the repression of embryonic properties after germination. Plant Physiol..

[21] Salaun C., Lepiniec L., Dubreucq B. (2021). Genetic and Molecular Control of Somatic Embryogenesis. Plants.

[22] Ikeda-Iwai M., Satoh S., Kamada H. (2002). Establishment of a reproducible tissue culture system for the induction of *Arabidopsis* somatic embryos. J. Exp. Bot..

[23] Kobayashi T., Nagayama Y., Higashi K., Kobayashi M. (2010). Establishment of a tissue culture system for somatic embryogenesis from germinating embryos of *Arabidopsis thaliana*. Plant Biotechnol..

[24] Luo Y., Koop H.S. (1997). Somatic embryogenesis in cultured immature zygotic embryos and leaf protoplasts of *Arabidopsis thaliana* ecotypes. Planta.

[25] Ikeda-Iwai M., Umehara M., Satoh S., Kamada H. (2003). Stress-induced somatic embryogenesis in vegetative tissues of *Arabidopsis thaliana*. Plant J..

[26] Su Y.H., Zhao X.Y., Liu Y.B., Zhang C.L., O’Neill S.D., Zhang X.S. (2009). Auxin-induced WUS expression is essential for embryonic stem cell renewal during somatic embryogenesis in *Arabidopsis*. Plant J..

[27] Bassuner B., Lam R., Lukowitz W., Yeung E. (2007). Auxin and root initiation in somatic embryos of *Arabidopsis*. Plant Cell Rep..

[28] Raghavan V. (2005). Control of leaf formation and somatic embryogenesis in cultured zygotic embryos of *Arabidopsis thaliana* by 2,4-dichlorophenoxyacetic acid (2,4-D). Isr. J. Plant Sci..

[29] Möller B., Weijers D. (2009). Auxin control of embryo patterning. Cold Spring Harb. Perspect. Biol..

[30] Friml J., Vieten A., Sauer M., Weijers D., Schwarz H., Hamann T., Offringa R., Jürgens G. (2003). Efflux-dependent auxin gradients establish the apical-basal axis of *Arabidopsis*. Nature.

[31] Heisler M., Ohno C., Das P., Sieber P., Reddy G., Long J., Meyerowitz E. (2005). Patterns of auxin transport and gene expression during primordium development revealed by live imaging of the *Arabidopsis* inflorescence meristem. Cur. Biol..

[32] Tautorus T.E., Fowke L.C., Dunstan D.I. (1991). Somatic embryogenesis in conifers. Can. J. Bot..

[33] Raza G., Singh M.B., Bhalla P.L. (2019). Somatic Embryogenesis and Plant Regeneration from Commercial Soybean Cultivars. Plants.

[34] Norgaard J.V., Krogstrup P. (1991). Cytokinin induced somatic embryogenesis from immature embryos of *Abies nordmanniana* Lk. Plant Cell Rep..

[35] San-José M.C., Corredoira E., Martínez M.T., Vidal N., Valladares S., Mallón R., Vieitez A.M. (2010). Shoot apex explants for induction of somatic embryogenesis in mature *Quercus robur* L. trees. Plant Cell Rep..

[36] Michalczuk L., Ribnicky D.M., Cooke T.J., Cohen J.D. (1992). Regulation of indole-3-acetic acid biosynthetic pathways in carrot cell cultures. Plant Physiol..

[37] Singh P., Sinha A.K., Dandekar T., Naseem M. (2017). Interplay between auxin and cytokinin and its impact on mitogen activated protein kinase (MAPK). Auxins and Cytokinins in Plant Biology: Methods and Protocols.

[38] Kotov A.A., Kotova L.M. (2018). Auxin-cytokinin interactions in regulating correlative inhibition in two-branched pea seedlings. J. Exp. Bot..

[39] Liao C.Y., Smet W., Brunoud G., Yoshida S., Vernoux T., Weijers D. (2015). Reporters for sensitive and quantitative measurement of auxin response. Nat. Meth..

[40] Jones B., Gunneras S.A., Petersson S.V., Tarkowski P., Graham N., May S., Dolezal K., Sandberg G., Ljung K. (2010). Cytokinin regulation of auxin synthesis in *Arabidopsis* involves a homeostatic feedback loop regulated via auxin and cytokinin signal transduction. Plant Cell.

[41] Chen S., Kurdyukov S., Kereszt A., Wang X., Gresshoff P., Rose R. (2009). The association of hoemeobox gene expression with stem cell formation and morphogenesis in cultured *Medicago truncatula*. Planta.

[42] Chen B., Fiers M., Dekkers B.J.W., Maas L., van Esse G.W., Angenent G.C., Zhao Y., Boutilier K. (2021). ABA signalling promotes cell totipotency in the shoot apex of germinating embryos. J. Exp. Bot..

[43] Stasolla C., van Zyl L., Egertsdotter U., Craig D., Liu W., Sederoff R. (2003). The effects of polyethylene glycol on gene expression of developing white spruce somatic embryos. Plant Physiol..

[44] Chakrabortee S., Boschetti C., Walton L.J., Sarkar S., Rubinsztein D.C., Tunnacliffe A. (2007). Hydrophilic protein associated with desiccation tolerance exhibits broad protein stabilization function. Proc. Natl. Acad. Sci. USA.

[45] Kvaalen H. (1994). Ethylene synthesis and growth in embryogenic tissue of Norway spruce–effects of oxygene, 1-aminocyclopropane-1-carboxylic acid, benzyladenine and 2,4-dichlorophenoxyacetic acid. Physiol. Plant.

[46] Kong L.S., Yeung E.C. (1995). Effects of silver-nitrate and polyethylene-glycol on white spruce (*Picea glauca*) somatic embryo development—Enhancing cotyledonary embryo formation and endogenous ABA content. Physiol. Plant.

[47] Gautier F., Eliášová K., Leplé J.C., Vondráková Z., Lomenech A.M., Le Metté C., Label P., Costa G., Trontin J.F., Teyssier C. (2018). Repetitive somatic embryogenesis induced cytological and proteomic changes in embryogenic lines of *Pseudotsuga menziesii* [Mirb.]. BMC Plant Biol..

[48] Ahmadi B., Shariatpanahi M.E., da Silva J.A.T. (2014). Efficient induction of microspore embryogenesis using abscisic acid, jasmonic acid and salicylic acid in *Brassica napus* L.. Plant Cell Tissue Organ Cult..

[49] Rudus I., Kepczynski J., Kepczynska E. (2001). The influence of the jasmonates and abscisic acid on callus growth and somatic embryogenesis in *Medicago sativa* L. tissue culture. Acta Physiol. Plant.

[50] Reinbothe C., Tewes A., Lehman J., Parthier B., Reinbothe S. (1994). Induction by methyl jasmonate of embryogenesis-related TED proteins and messenger RNAs in *Nicotiana plumbaginifolia*. Plant Sci..

[51] Wilen R.W., van Rooijen G.J.H., Pearce D.W., Pharis R.P., Holbrook L.A., Moloney M.M. (1991). Effects of jasmonic acid on embryo-specific processes in *Brassica* and *Linum* oilseeds. Plant Physiol..

[52] Mira M.M., Wally O.S., Elhiti M., El-Shanshory A., Reddy D.S., Hill R.D., Stasolla C. (2016). Jasmonic acid is a downstream component in the modulation of somatic embryogenesis by *Arabidopsis* Class 2 phytoglobin. J. Exp. Bot..

[53] Loyola-Vargas V.M., Ochoa-Alejo N., Loyola-Vargas V.M., Ochoa-Alejo N. (2016). Somatic embryogenesis. An overview. Somatic Embryogenesis. Fundamental Aspects and Applications.

[54] Ayil-Gutiérrez B.A., Galaz-Ávalos R.M., Peña-Cabrera E., Loyola-Vargas V.M. (2013). Dynamics of the concentration of IAA and some of its conjugates during the induction of somatic embryogenesis in *Coffea canephora*. Plant Signal. Behav..

[55] Elhiti M., Hebelstrup K.H., Wang A., Li C., Cui Y., Hill R.D., Stasolla C. (2013). Function of type-2 *Arabidopsis* hemoglobin in the auxin-mediated formation of embryogenic cells during morphogenesis. Plant J..

[56] Teale W., Paponov I., Palme K. (2006). Auxin in action: Signalling, transport and the control of plant growth and development. Nat. Rev. Mol. Cell Biol..

[57] Riechmann J.L., Heard J., Martin G., Reuber L., Jiang C.Z., Keddie J., Adam L., Pineda O., Ratcliffe O.J., Samaha R.R. (2000). *Arabidopsis* transcription factors: Genome-wide comparative analysis among eukaryotes. Science.

[58] Rademacher E.H., Moller B., Lokerse A.S., Llavata-Peris C.I., Berg W., Weijers D.A. (2011). Cellular expression map of the *Arabidopsis AUXIN RESPONSE FACTOR* gene family. Plant J..

[59] Wójcikowska B., Gaj M.D. (2017). Expression profiling of *AUXIN RESPONSE FACTOR* genes during somatic embryogenesis induction in *Arabidopsis*. Plant Cell Rep.

[60] Gliwicka M., Nowak K., Balazadeh S., Mueller-Roeber B., Gaj M.D. (2013). Extensive modulation of the transcription factor transcriptome during somatic embryogenesis in *Arabidopsis thaliana*. PLoS ONE.

[61] Elhiti M., Stasolla C., Wang A. (2013). Molecular regulation of plant somatic embryogenesis. Vitr. Cell Dev. Biol.-Plant.

[62] Orozco-Cardenas M.L., Ryan C.A. (1999). Hydrogen peroxide is generated systemically in plant leaves by wounding and systemin via the octadecanoid pathway. Proc. Natl. Acad. Sci. USA.

[63] Kairong C., Ji L., Gengmei X., Jianlong L., Lihong W., Yafu W. (2002). Effect of hydrogen peroxide on synthesis of proteins during somatic embryogenesis in *Lycium barbarum*. Plant Cell Tissue Organ Cult..

[64] Rose R.J., Mantiri F.R., Kurdyukov S., Chen S.K., Wang X.D., Nolan K.E., Sheahan M.B., Pua E., Davey M. (2010). Developmental Biology of Somatic Embryogenesis. Plant Developmental Biology—Biotechnological Perspectives.

[65] Thibaud-Nissen F., Shealy R.T., Khanna A., Vodkin L.O. (2003). Clustering of microarray data reveals transcript patterns associated with somatic embryogenesis in soybean. Plant Physiol..

[66] Kuczak M., Kurczyńska E. (2020). Cell wall composition as a marker of the reprogramming of the cell fate on the example of a *Daucus carota* (L.) hypocotyl in which somatic embryogenesis was induced. Int. J. Mol. Sci..

[67] Magnani E., Jiménez-Gómez J.M., Soubigou-Taconnat L., Lepiniec L., Fiume E. (2017). Profiling the onset of somatic embryogenesis in *Arabidopsis*. BMC Genom..

[68] Ikeuchi M., Sugimoto K., Iwase A. (2013). Plant callus: Mechanisms of induction and repression. Plant Cell.

[69] Galiba G., Yamada Y. (1988). A novel method increasing the frequency of somatic embryogenesis in wheat tissue culture by NaCl and KCl supplementation. Plant Cell Rep..

[70] Kamada H., Ishikawa K., Saga H., Harada H. (1993). Induction of somatic embryogenesis in carrot by osmotic stress. Plant Tissue Cult. Lett..

[71] Stasolla C. (2010). Glutathione redox regulation of in vitro embryogenesis. Plant Physiol. Biochem..

[72] Al-Khayri J.M., Al-Bahrany A.M. (2012). Effect of abscisic acid and polyethylene glycol on the synchronization of somatic embryo development in date palm (*Phoenix dactylifera* L.). Biotechnology.

[73] Akula A., Akula C., Bateson M. (2000). Betaine a novel candidate for rapid induction of somatic embryogenesis in tea (*Camellia sinensis* (L.) O. Kuntze). Plant Growth Regul..

[74] Ladyman J.A.R., Girard B. (1992). Cucumber somatic embryo development on various gelling agents and carbohydrate sources. HortScience.

[75] Valencia-Lozano E., Ibarra J.E., Herrera-Ubaldo H., De Folter S., Cabrera-Ponce J.L. (2021). Osmotic stress-induced somatic embryo maturation of coffee *Coffea arabica* L., shoot and root apical meristems development and robustness. Sci. Rep..

[76] Lichter R. (1982). Induction of haploid plants from isolated pollen of *Brassica napus*. Z. Pflanzenphysiol..

[77] Joosen R., Cordewener J., Supena E.D.J., Vorst O., Lammers M., Maliepaard C., Zeilmaker T., Miki B., America T., Custers J. (2007). Combined transcriptome and proteome analysis identifies pathways and markers associated with the establishment of rapeseed microspore-derived embryo development. Plant Physiol..

[78] Dubas E., Moravciková J., Libantová J., Matušíková I., Benková E., Zur I., Krzewska M. (2014). The influence of heat stress on auxin distribution in transgenic *B. napus* microspores and microspore-derived embryos. Protoplasma.

[79] Decout E., Dubois T., Guedira M., Dubois J., Audran J.-C., Vasseur J. (1994). Role of temperature as a triggering signal for organogenesis or somatic embryogenesis in wounded leaves of chicory cultured in vitro. J. Exp. Bot..

[80] Touraev A., Vicente O., Heberle-Borse E. (1997). Initiation of embryogenesis by stress. Trends Plant Sci..

[81] Castander-Olarieta A., Pereira C., Montalbán I.A., Mendes V.M., Correia S., Suárez-Álvarez S., Manadas B., Canhoto J., Moncaleán P. (2021). Proteome-wide analysis of heat-stress in *Pinus radiata* somatic embryos reveals a combined response of sugar metabolism and translational regulation mechanisms. Front. Plant Sci..

[82] Elhiti M., Huang S., Mira M.M., Hill R.D., Stasolla C. (2018). Redirecting cell fate during in vitro embryogenesis: Phytoglobins as molecular switches. Front. Plant Sci..

[83] Greenberg J.T. (1996). Programmed cell death: A way of life for plants. Proc. Natl. Acad. Sci. USA.

[84] Filonova L.H., von Arnold S., Daniel D., Bozhkov P.B. (2002). Programmed cell death eliminates all but one embryo in a polyembryonic plant seed. Cell Death Differ..

[85] Kundu M., Thompson C.B. (2005). Macroautophagy versus mitochondrial autophagy: A question of fate?. Cell Death Differ..

[86] Huang S., Hill R.D., Wally O.S., Dionisio G., Ayele B.T., Jami S.K., Stasolla C. (2014). Hemoglobin control of cell survival/death decision regulates in vitro plant embryogenesis. Plant Physiol..

[87] Smertenko A.P., Bozhkov P.V., Filonova L.H., von Arnold S., Hussey P.J. (2003). Reorganization of the cytoskeleton during developmental programmed cell death in *Picea abies* embryos. Plant J..

[88] Varnier A.L., Mazeyrat-Gourbeyre F., Sangwan R.S., Clément C. (2005). Programmed cell death progressively models the development of anther sporophytic tissues from the tapetum and is triggered in pollen grains during maturation. J Struct. Biol..

[89] Varnier A.L., Jacquard C., Clement C., Touraev A., Foster B.P., Jain S.M. (2009). Programmed cell death and microspore embryognesis. Advances in Haploid Production in Higher Plants.

[90] Jin F., Hu L., Yuan D., Xu J., Gao W., He L., Yang X., Zhang X. (2014). Comparative transcriptome analysis between somatic embryos (SEs) and zygotic embryos in cotton: Evidence for stress response functions in SE development. Plant Biotechnol. J..

[91] Che P., Lall S., Howell S.H. (2007). Developmental steps in acquiring competence for shoot development in *Arabidopsis* tissue culture. Planta.

[92] De Jong A.J., Cordewener J., LoSchiavo F., Terzi M., Vandekerckhove J., Van Kammen A., De Vries S.C. (1992). A carrot somatic embryo mutant is rescued by chitinase. Plant Cell.

[93] Zheng Q., Zheng Y., Perry S.E. (2013). AGAMOUS-Like15 promotes somatic embryogenesis in *Arabidopsis thaliana* and *Glycine max* in part by control of ethylene biosynthesis and response. Plant Physiol..

[94] Salvo S.A., Hirsch C.N., Buell C.R., Kaeppler S.M., Kaeppler H.F. (2014). Whole transcriptome profiling of maize during early somatic embryogenesis reveals altered expression of stress factors and embryogenesis-related genes. PLoS ONE.

[95] Janosevic D., Budimir S. (2006). Shoot apical meristem structure and *STM* expression in *has* mutant of *Arabidopsis thaliana*. Biol. Plant.

[96] Cole M., Nolte C., Werr W. (2006). Nuclear import of the transcription factor *SHOOT MERISTEMLESS* depends on heterodimerization with BLH proteins expressed in discrete sub-domains of the shoot apical meristem of *Arabidopsis thaliana*. Nucleic Acids Res..

[97] Sharma V., Fletcher J. (2002). Maintenance of shoot and floral meristem cell proliferation and fate. Plant Physiol..

[98] Lenhard M., Jurgens G., Laux T. (2002). The *WUSCHEL* and *SHOOTMERISTEMLESS* genes fulfil complementary roles in *Arabidopsis* shoot meristem regulation. Development.

[99] Mayer K.F., Schoof H., Haecker A., Lenhard M., Jurgens G., Laux T. (1998). Role of *WUSCHEL* in regulating stem cell fate in the *Arabidopsis* shoot meristem. Cell.

[100] Ikeda M., Mitsuda N., Ohme-Takagi M. (2009). *Arabidopsis WUSCHEL* is a bifunctional transcription factor that acts as a repressor in stem cell regulation and as an activator in floral patterning. Plant Cell.

[101] Laux T., Mayer K.F., Berger J., Jurgens G. (1996). The *WUSCHEL* gene is required for shoot and floral meristem integrity in *Arabidopsis*. Development.

[102] Yadav R.K., Perales M., Gruel J., Girke T., Jonsson H., Reddy G.V. (2011). WUSCHEL protein movement mediates stem cell homeostasis in the *Arabidopsis* shoot apex. Genes Dev..

[103] Zhang T.Q., Lian H., Zhou C.M., Xu L., Jiao Y., Wang J.W. (2017). A two-step model for de novo activation of WUSCHEL during plant shoot regeneration. Plant Cell.

[104] Zuo J., Niu Q.W., Frugis G., Chua N.H. (2002). The *WUSCHEL* gene promotes vegetative-to-embryonic transition in *Arabidopsis*. Plant J..

[105] Rashid S., Yamaji N., Kyo M. (2007). Shoot formation from root tip region: A developmental alteration by *WUS* in transgenic tobacco. Plant Cell Rep..

[106] Arroyo-Herrera A., Ku-Gonzalez A., Canche-Moo R., Quiroz-Figueroa F.R., Loyola-Vargas V.M., Rodriguez-Zapata L.C., Castino E. (2008). Expression of *WUSCHEL* in *Coffea canephora* causes ectopic morphogenesis and increases somatic embryogenesis. Plant Cell Tissue Organ Cult..

[107] Zheng W., Zhang X., Yang Z., Wu J., Li F., Duan L., Liu C., Lu L., Zhang C., Li F. (2014). AtWuschel promotes formation of the embryogenic callus in *Gossypium hirsutum*. PLoS ONE.

[108] Lowe K., La Rota M., Hoerster G., Hastings C., Wang N., Chamberlin M., Wu E., Jones T., Gordon-Kamm W. (2018). Rapid genotype “independent” *Zea mays* L. (maize) transformation via direct somatic embryogenesis. Vitr. Cell Dev. Biol.-Plant.

[109] Gaj M.D., Zhang S.B., Harada J.J., Lemaux P.G. (2005). Leafy cotyledon genes are essential for induction of somatic embryogenesis of *Arabidopsis*. Planta.

[110] Lotan T., Ohto M., Yee K.M., West M.A.L., Lo R., Kwong R.W., Harada J.J. (1998). *Arabidopsis LEAFY COTYLEDON1* is sufficient to induce embryo development in vegetative cells. Cell.

[111] Luerssen H., Kirik V., Herrmann P., Miséra S. (1998). *FUSCA3* encodes a protein with a conserved VP1/ABI3-like B3 domain which is of functional importance for the regulation of seed maturation in *Arabidopsis thaliana*. Plant J..

[112] Uddenberg D., Valladares S., Abrahamsson M. (2011). Embryogenic potential and expression of embryogenesis-related genes in conifers are affected by treatment with a histone deacetylase inhibitor. Planta.

[113] Zhang Y., Clemens A., Maximova S.N., Guiltinan M.J. (2014). The *Theobroma cacao* B3 domain transcription factor *TcLEC2* plays a dual role in control of embryo development and maturation. BMC Plant Biol..

[114] Kim H.U., Jung S.J., Lee K.R., Kim E.H., Lee S.M., Roh K.H., Kim J.B. (2014). Ectopic overexpression of castor bean *LEAFY COTYLEDON2* (*LEC2*) in *Arabidopsis* triggers the expression of genes that encode regulators of seed maturation and oil body proteins in vegetative tissues. FEBS Open Bio.

[115] Ledwon A., Gaj M. (2009). *LEAFY COTYLEDON2* gene expression and auxin treatment in relation to embryogenic capacity of *Arabidopsis* somatic cells. Plant Cell Rep..

[116] Brand A., Quimbaya M., Tohme J., Chavarriaga-Aguirre P. (2019). *Arabidopsis**LEC1* and *LEC2* orthologous genes are key regulators of somatic embryogenesis in cassava. Front. Plant Sci..

[117] Boutilier K., Offringa R., Sharma V.K., Kieft H., Ouellet T., Zhang L., Hattori J., Liu C.-M., van Lammeren A.M., Miki B.M. (2002). Ectopic expression of *BABY BOOM* triggers a conversion from vegetative to embryonic growth. Plant Cell.

[118] Krizek B.A. (2015). *AINTEGUMENTA-LIKE* genes have partly overlapping functions with *AINTEGUMENTA* but make distinct contributions to *Arabidopsis thaliana* flower development. J. Exp. Bot..

[119] Galinha C., Hofhuis H., Luijten M., Willemsen V., Blilou I., Heidstra R., Scheres B. (2007). PLETHORA proteins as dose-dependent master regulators of *Arabidopsis* root development. Nature.

[120] Aida M., Beis D., Heidstra R., Willemsen V., Blilou I., Galinha C., Nussaume L., Noh Y.S., Amasino R., Scheres B. (2004). The PLETHORA genes mediate patterning of the *Arabidopsis* root stem cell niche. Cell.

[121] Kulinska-Lukaszek K., Tobojka M., Adamiok A., Kurczynska E. (2012). Expression of the BBM gene during somatic embryogenesis of *Arabidopsis thaliana*. Biol. Plant.

[122] Deng W., Luo K., Li Z., Yang Y. (2009). A novel method for induction of plant regeneration via somatic embryogenesis. Plant Sci..

[123] Srinivasan C., Liu Z., Heidmann I., Supena E.D., Fukuoka H., Joosen R., Lambalk J., Angenent G., Scorza R., Custers J.B. (2007). Heterologous expression of the *BABY BOOM* AP2/ERF transcription factor enhances the regeneration capacity of tobacco (*Nicotiana tabacum* L.). Planta.

[124] Florez S.L., Erwin R.L., Maximova S.N., Guiltinan M.J., Curtis W.R. (2015). Enhanced somatic embryogenesis in *Theobroma cacao* using the homologous *BABY BOOM* transcription factor. BMC Plant Biol..

[125] Silva A.T., Barduche D., do Livramento K.G., Paiva L.V. (2015). A putative *BABY BOOM-like* gene (*CaBBM*) is expressed in embryogenic calli and embryogenic cell suspension culture of *Coffea arabica* L.. Vitr. Cell. Dev. Biol.-Plant.

[126] Nic-Can G.I., López-Torres A., Barredo-Pool F., Wrobel K., Loyola-Vargas V.M., Rojas-Herrera R., De-la-Peña C. (2013). New insights into somatic embryogenesis: *LEAFY COTYLEDON1*, *BABY BOOM1* and *WUSCHEL-RELATED HOMEOBOX4* are epigenetically regulated in *Coffea canephora*. PLoS ONE.

[127] Lowe K., Wu E., Wang N., Hoerster G., Hastings C., Cho M.J., Scelonge C., Lenderts B., Chamberlin M., Cushatt J. (2016). Morphogenic Regulators *Baby boom* and *Wuschel* improve monocot transformation. Plant Cell.

[128] Schmidt E., Guzzo F., Toonen M., De Vries S. (1997). A leucine-rich repeat containing receptor-like kinase marks somatic plant cells competent to form embryos. Development.

[129] Hecht V., Vielle-Calzada J.-P., Hartog M.V., Schmidt E.D.L., Boutilier K., Grossniklaus U., de Vries S.C. (2001). The *Arabidopsis Somatic Embryogenesis Receptor Kinase 1* gene is expressed in developing ovules and embryos and enhances embryogenic competence in culture. Plant Physiol..

[130] Nolan K.E., Irwanto R.R., Rose R.J. (2003). Auxin up-regulates *MtSERK1* expression in both *Medicago truncatula* root-forming and embryogenic cultures. Plant Physiol..

[131] Singla B., Khurana J.P., Khurana P. (2009). Structural characterization and expression analysis of the *SERK/SERL* gene family in rice (*Oryza sativa*). Int. J. Plant Genom..

[132] Singh A., Khurana P. (2017). Ectopic expression of *Triticum aestivum SERK* genes (*TaSERKs*) control plant growth and development in *Arabidopsis*. Sci. Rep..

[133] Hill R.D. (2012). Non-symbiotic haemoglobins–What’s happening beyond nitric oxide scavenging?. AoB Plants.

[134] Stasolla C., Hill R.D. (2017). Determining cellular responses: Phytoglobins may direct the traffic. Trends Plant Sci..

[135] Mira M.M., Huang S., Kapoor K., Hammond C., Hill R.D., Stasolla C. (2017). Expression of *Arabidopsis* class 1 phytoglobin (AtPgb1) delays death and degradation of the root apical meristem during severe PEG-induced water deficit. J. Exp. Bot..

[136] DeLille J.M., Sehnke P.C., Ferl R.J. (2001). The *Arabidopsis* 14-3-3 family of signaling regulators. Plant Physiol..

[137] Zhang Z.T., Zhou Y., Li Y., Shao S.Q., Li B.Y., Shi H.Y. (2010). Interactome analysis of the six cotton 14-3-3s that are preferentially expressed in fibres and involved in cell elongation. J. Exp. Bot..

[138] Konagaya K.I., Matsushita Y., Kasahara M., Nyunoya H. (2004). Members of 14-3-3 protein isoforms interacting with the resistance gene product N and the elicitor of *Tobacco mosaic virus*. J. Gen. Plant Pathol..

[139] Camoni L., Visconti S., Aducci P., Marra M. (2018). 14-3-3 Proteins in plant hormone signaling doing several things at once. Front. Plant Sci..

[140] Schoonheim P.J., Sinnige M.P., Casaretto J.A., Veiga H., Bunney T.D., Quatrano R.S. (2007). 14-3-3 adaptor proteins are intermediates in ABA signal transduction during barley seed germination. Plant J..

[141] Yao Y., Du Y., Jiang L., Liu J.Y. (2007). Molecular analysis and expression patterns of the 14-3-3 gene family from *Oryza sativa*. BMB Rep..

[142] Michniewicz M., Zago M.K., Abas L., Weijers D., Schweighofer A., Meskiene I. (2007). Antagonistic regulation of PIN phosphorylation by PP2A and PINOID directs auxin flux. Cell.

[143] Marsoni M., Bracale M., Espen L., Prinsi B., Negri A., Vannini C. (2008). Proteomic analysis of somatic embryogenesis in *Vitis vinifera*. Plant Cell Rep..

[144] Lyngved R., Renaut J., Hausman J.F., Iversen T.H., Hvoslef-Eide A.K. (2008). Embryo-specific proteins in *Cyclamen persicum* analyzed with 2-D DIGE. J. Plant Growth Regul..

[145] Méndez-Hernández H.A., Ledezma-Rodríguez M., Avilez-Montalvo R.N., Juárez-Gómez Y.L., Skeete A., Avilez-Montalvo J., De-la-Peña C., Loyola-Vargas V.M. (2019). Signaling overview of plant somatic embryogenesis. Front. Plant Sci..

[146] Janssens V., Goris J. (2001). Protein phosphatase 2A: A highly regulated family of serine/threonine phosphatases implicated in cell growth and signalling. Biochem. J..

